# New arenas of inclusion: experiences of individuals with intellectual disability employed in higher education

**DOI:** 10.3389/fpsyt.2025.1640613

**Published:** 2025-08-14

**Authors:** Gro H. Ramsdal, Silje Mevold, Leif Inge Johansen, Rolf Wynn

**Affiliations:** ^1^ Department of Social Education, Faculty of Health Sciences, UiT The Arctic University of Norway, Tromsø, Norway; ^2^ Department of Clinical Medicine, Faculty of Health Sciences, UiT The Arctic University of Norway, Tromsø, Norway

**Keywords:** intellectual disabililties, neurodevelopmental disorders, inclusion, non-sheltered employment, interview study

## Abstract

**Introduction:**

Prior studies have found that people with intellectual disabilities (ID) often experience being excluded from important parts of society, and unemployment and lack of social connections may severely negatively impact quality of life for people with ID.

**Methods:**

Five persons with ID participating in a project at a Norwegian University were interviewed, with a particular emphasis on how they experienced social inclusion at this non-sheltered work environment. The participants also worked in a sheltered environment. The data analysis was performed in accordance with the method of thematic analysis.

**Results:**

Three main themes that emerged from the data: 1) ‘Experienced employees’, indicating that the participants had worked in different types of jobs and evaluated their current work positively in light of prior work experiences; 2) ‘Suitable work challenges’, implying that the participants described having had some influence over their work and were generally satisfied with their assignments; 3) ‘Social belonging’, demonstrating how the participants underlined the importance of meeting new people, expanding their network and being appreciated at work.

**Discussion:**

The participants were generally satisfied with work at the University, and reported feeling included through the forming of relationships with other people at the workplace. However, they also emphasised the importance of their work in a sheltered environment, where they felt secure and had friends.

**Conclusion:**

The participant described experiences of belonging and being included when at work at the University. A combination of sheltered and non-sheltered work seemed to be experienced positively by the participants.

## Introduction

The marginal position of people with ID has been well documented (1–3). In a review of empirical findings, people with ID were reported to have 3–4 times higher unemployment rates than non-disabled peers and more likely to be employed in sheltered or segregated settings (4). Furthermore, they were less likely to be involved in community participation and group leisure activities and had an average social network of 3.1 persons, whereof one was usually a professional service or staff member. Lack of social connections and appropriate activities seem to be among the major concerns of people with ID (3, 5). In a study of young adults with ID, the participants reported feeling left out. They experienced trouble finding employment and were often bored (3). These studies indicate a substantial participation gap between people with and without ID. Participation is defined by the World Health Organization (WHO) (6) as ‘the performance of people in actual activities in social life domains through interaction with others in the context in which they live’. Four social life domains are included when assessing this type of participation, namely (1) domestic life (2) interpersonal life (3) major life arenas like education and work and (4) community, civic and social life. The question is whether the participation of people with ID in the ‘actual activities in social life domains with others in the context in which they live’ (6), constitutes genuine participation or mere exposure to the general community without actually being *socially integrated*.

Some researchers discuss this issue emphasizing the notion that when people living segregated lives are encouraged to expose themselves to arenas in the general community, they must benefit from such exposure (7). A Swedish researcher found that exposing disabled children to regular classrooms did not result in the presumed contacts and friendships between disabled and non-disabled pupils (8). It has been argued that there are segregating mechanisms in the normal social interaction that these integration projects did not address (8). Studying children with milder ID in special schools and after school programs, another Swedish researcher (9) found that disabled children were overshadowed by the nondisabled children and never really participated on their own terms in the sense that non-disabled children set the standards, made the choices and took the initiatives when playing together. Consequently, other researchers (7) maintain that service providers cannot automatically assume that people with ID prefer integration before segregation in all arenas or situations. They call to attention that overexposure or exposure to hostile social environments may be harmful. This argumentation may be misused to focus attention on the need to protect intellectually disabled people from community participation due to their vulnerability, and thus withholding the privilege of choice, exploration, and self-determination from them. However, in sum, these researchers point to the fact that there is a need to know more about the experiences of people with disability concerning their community participation processes (7).

Studies on the *effect* of community participation seem to be limited although such participation is an aspect of social inclusion that has been found to contribute to the quality of life or wellbeing of people with ID (10–14). Nevertheless, there is a knowledge gap as to the experiences of people with ID when it comes to the effects and consequences of community participation in general and in some arenas in particular, and further studies are needed to expand our knowledge of these experiences.

It has also been argued that community participation is essential due to the increased risk of discrimination and denigration for those who did not participate in socially valued roles (15). Community participation implies shared activities in ordinary settings (16) and thus being enabled to access community resources and experiencing not only physical inclusion but also *social inclusion* in the form of relationships, membership, and belonging (17). Belonging, according to one researcher (16, p.16) ‘is often described as having elements of intimacy, connectedness, membership, commitment, solidarity and reciprocity’. Although belonging is presumed to be a universal need (18) it has received surprisingly little attention within the disability literature (16). Nevertheless, it is known that when people with ID participate by being present in the community, they seem to have a better possibility, through repeated encounters, to be recognized by others and to develop acquaintances and attachments to people who spend time in the same places (19). Presence in the community can create new informal memberships and change the expectations of other community members. Thus, *the interaction* of people with and without ID in itself is considered a presupposition for inclusion and belonging and therefore make *presence* critical to belonging. However, inclusion (as in presence), although providing the foundation for belonging, is not a guarantee for the development of such supportive relationships. Something more is needed, and this can be defined as the experience of being present, invited, welcomed, known, accepted, supported, heard, befriended, needed and loved (16).

According to the WHO, one of the four life arenas defining community participation is education and work (6). Unfortunately, in the US there seems to be a steadily growing tendency to offer segregated and non-work day programs to people with significant disabilities (20). This development continues in spite of research indicating that most people with disabilities and their families prefer competitive integrated employment (CIE) to segregated employment or day services (21–23). One systematic review showed that people with ID experience higher rates of job satisfaction in integrated employment (24). Another study found that in most programs, participants spent only half their day in purposeful activities and sometimes spent part of their time in age-inappropriate activities (25). Furthermore, a recent systematic review suggested that people with ID working in segregated employment are not more likely to obtain competitive employment (23). Models that promote CIE such as supported and customized employment on the other hand, were found to result in improved outcomes in key domains like economy, quality of life and mental health. However, in depth studies of the experiences of people with ID regarding work are scarce (26). So even though people with ID indicate that they prefer not to be in sheltered employment, little research specifically examines the pathways of how they prefer to access such employment, concludes the authors of one systematic review, pointing out that this lack of knowledge is a barrier to improve employment outcomes for people with ID (23).

### Barriers of inclusion and belonging

A limited number of studies have been aimed at entangling which environmental factors have positive or negative effects on community participation. A systematic review (27) identified the lack of transportation and not feeling accepted as the main environmental factors negatively influencing community participation and inclusion. People with ID themselves in addition identify *lack of acceptance* and negative attitudes to be among the most important barriers to social inclusion (1). People with ID want to participate and being treated as individuals not defined by their disabilities requires an accepting environment (28). Young adults with ID described acceptance as when people initiated conversations with them and treated them with respect like any other person as opposed to being left out, ignored or treated differently (29). The most effective way of promoting such acceptance is personal contact (16). Personal contact, however, is hampered by the lack of community participation (4).

As described above, presence in various community arenas is necessary but not sufficient to experience inclusion in the form of belonging, for people with ID. It has been argued that, most people with ID cannot or do not wish to seek out the *traditional* routes to social inclusion, like competitive employment and independent living (29). Still, they do want to be ‘attached’ and to ‘belong’. Pursuing their presence in the general community can be influential in fostering a sense of belonging (16) and among the four arenas important for community participation, WHO has identified education and work to be one of them (6). Nevertheless, in some educational and work arenas people with ID are almost totally absent, for example at most universities. Higher education is a high-status arena in modern communities and presence in this arena might contribute to increased acceptance in society and provide experiences of belonging to people with ID (16, 30). Because few people with ID are employed in university settings, research on such inclusion processes are very hard to find. Due to this gap of knowledge, there is a need to study how people with ID describe, master and react to the inclusion processes involved in entering this to them new and non-sheltered arena of employment. There is a need to study the experiences of people with ID in non-sheltered employment to learn more about whether and how they succeed in finding a sense of belonging in such work arenas. The exploration of these experiences is the main focus of the current study.

### Background of the project

All four authors of this article are employed at The Faculty of Health Sciences at the Arctic University of Norway (UiT), three of the researchers at the Department of Social Education, a Department with a strong focus on disability in general and ID in particular. This Department has during several years worked to develop a project enabling people with disability in general and ID in particular to find work assignments in higher education, thus enabling them to *be present* and *develop belonging* at the university, without being students or full-time employees. Developing such a project implied overcoming many administrative barriers in order to remain within the organizational frame of the institution. Gradually, the Department was first able to offer teaching assignments to 6 people with ID (30) and finally could offer other work assignments to 5 adults with disabilities, mostly ID. When the participants had been employed for about one year, they were invited to participate in a study exploring their experiences with these work assignments, and all five employees gave their consent to being interviewed about these experiences.

The aim of the study was to explore the experiences of the study participants. The researchers wanted to examine what the participants themselves experienced as important barriers to their inclusion processes. The researchers also wanted to explore factors that the participants thought contributed to their inclusion processes. In addition, the researchers wanted to explore whether these inclusion processes resulted in personal benefits to the participants such as the development of a sense of belonging at the university.

## Materials and methods

### Methodological approach

The researchers followed the general ethical guidelines from the National Research Ethics Committees in the process of recruitment, consent, data collection and publication (31) and the study received approval by the Norwegian Centre for Research Data (NSD project no. 454008). All participants gave oral and written consent to participate in the study. The study was based on qualitative interview data involving five adult participants with neurodevelopmental disabilities (mostly ID) who talked about their experiences regarding their work assignments at the University.

Although the researchers did experience some communicative challenges in these interviews and are aware that this constitutes a vulnerability, the researchers in this study agree with other researchers maintaining that it is essential to include people with ID in research about their inclusion processes (32). Only by talking to them will one be able to gain knowledge about this group’s work life experiences. Furthermore, if vulnerable groups are not included in research, one may risk violating the principle of equal treatment as described in the Convention on the Rights of Persons with Disabilities (CRPD) (33). Thus, it is a positive trend that, there are more studies concerning the living conditions of vulnerable groups, their work experiences and general well-being and that they are found to appreciate being given the opportunity to ‘let their voices be heard’ (34) (35, p. 216). Taking these various arguments and knowledges into consideration, the researchers of this study concluded that interviewing persons with ID is necessary to understand their experiences with inclusion in various life arenas.

### Recruitment

Among the 5 individuals with neurodevelopmental disabilities employed at the Department of Social Education at UiT at that time, all were invited to participate in the research interviews. Their participation in the work inclusion project was the only inclusion criterion. The participants were employed to perform working assignments at the Department one day a week so that only one participant was present at the Department at the same time. One of the researchers talked with each of the employees. The research project was explained, and the employees were asked if they were interested in participating in the research interviews. Some of them had already participated in other research interviews and thus knew what this type of participation implied. They were all given two to three weeks to talk with their friends and family before deciding on participation. During this period, they were provided with oral and written information about how the interviews would be performed and were repeatedly reminded that participation was voluntary and that they could withdraw their consent at any time without any consequences, and that their answers would remain anonymous. The researcher that recruited the participants was familiar to them as they had become acquainted when sharing their working environment once a week. All five employees willingly gave their consent and seemed rather excited to participate in the research interviews.

In this study, there was an increased risk of being recognised because this particular group of employees is relatively small. The participants were therefore informed that even though the researchers did everything they could to anonymise the data, there was still a certain risk that someone might recognise them. This aspect of the study was also discussed thoroughly and repeatedly with those performing the assessment of the project at the Norwegian Centre for Research Data.

### The participants

The participants were two men and three women between the age of 25 and 40 who had neurodevelopmental disorders. They all had diverse experiences from sheltered employment, like Permanently Adapted Work (PAW). The participants had experiences with various types of jobs, some long-term employment and others with more short-term employments. This means that they were able to function quite independently in many respects, as in activities of daily living but they were also capable of using public transport and to participate independently in sports tournaments in neighbouring towns, Yet, their capacity for working independently did not make a regular job attainable. Nevertheless, they had a capacity for work that made them able to take supported employment in jobs that were not too physically or cognitively challenging. All had neurodevelopmental disorders, mainly ID. Most participants lived in some form of adapted housing, and some lived with their parents. When the University recruited them for the inclusion project, they were all in supported employment received disability benefits and additionally they received bonus wages from working in supported employment. They were full time employed in PAW and had previous work experience from different assignments such as office cleaning, and catering. When joining the inclusion project at the university, participants were offered through their present workplace to work one of their working days at UiT, keeping their present wages and remain the rest of their working days with their original employer in PAW. This meant that each work day only one of the five participants had work assignments and lunch with the staff at the Department, providing a possibility to develop inclusion and belonging in ordinary working life. The participants also took part in birthday celebrations, Easter and Christmas lunches, lunch concerts, the Department’s summer break celebration, and other social events taking place on the work day when they were present and had work assignments at the University.

### The interviews

In this study, qualitative semi-structured interviews were performed. The questions in the interview guide were open-ended and allowed for follow-up questions. Questions tapped the participants’ experiences with working one day a week at the University and in particular their experiences of inclusion and belonging in their work environment at the University. The interview guide was organized into four main topics, the first topic was ‘Background experiences’, where they were asked to talk about themselves and about prior work experience. The interviewer also asked about their ‘dream job’ and what kind of activities they liked in general. The second topic was ‘The University job assignment’, tapping into how they got the job at UiT and what their assignments were and how they liked them. Furthermore, the interviewer asked about what was difficult and what was fun about the assignments, if they got feed-back regarding the job they did and who they turned to if they needed help with their assignments, if they wanted more or less assignments and if they could make their own decisions at work and influence the way their tasks were performed. The third topic was called ‘Equality’, tapping more directly into inclusion and belonging asking open questions about how they were received and treated by their colleagues at the Department. Subsequently, the interviewer suggested specific possible response categories such as if the colleagues were busy, dismissive, irritated, helpful, friendly or respectful. These suggestions were added to make negative answers more normal, expected and ‘allowed’, as it is known that people are reluctant or afraid to criticize their employers. Positive words were also eventually suggested because this was an abstract question and giving examples of answers could improve the understanding of the question. Finally, the fourth topic was called ‘Communication’, tapping specifically into the development of belonging through questions about how they experienced their relationships at their new job and some questions explored how they *understood* and *categorized* the resulting relationships, like friendships, acquaintances or like colleagues.

The participants were interviewed in a conference room at the University and were offered warm and cold beverages. The interviews lasted from 45 minutes to one hour. All were offered to take a break during the interview but only one of the participants accepted this offer.

Unfortunately, the researchers were unable to include individuals with ID in the research team, because the individuals with the relevant experience were all included in the planned research sample. Nevertheless, after the analysis process was completed, member checking was performed by reinterviewing three of the five participants to examine if the researchers had understood the participants accurately in the sense that the researchers had perceived their communicational content the way it was intended. These were the three participants who agreed to a second interview. About forty-five minutes were spent with each participant, first explaining that the researchers wanted to be sure that they had not misunderstood what the participants meant to say. In short, simple sentences were used to describe the content of what the researchers had understood the participants had said at the interview and then they were asked if that was what they had meant to say. For instance, the researcher would say: ‘At the interview you said you liked the work assignments at the Department. Did I get this right?’ Then they were asked if this experience had changed, as several months had passed since they were interviewed. The researchers also actively explored the possibility of more negative experiences communicating that that would be interesting and ok to talk about. The participants expressed enthusiasm about the fact that the researchers wanted to interview them again and they were unequivocally clear that the themes emerging from the analysis represented what they had been saying in the interviews. They also confirmed that their experiences with the work assignments at the Department had not changed during the following months. More details regarding the interview questions can be found in **Table 1**.

**Table 1:** Examples of interview questions.

1 Background questions
‘Can you tell me a bit about yourself?’ ‘What jobs have you had before coming to the University?’, ‘What kind of job do you do at `name of supported employment firm?’ ‘How do you like your job outside UiT?’ ‘What is your dream job?’ ‘What kind of activities do you like’
2 The University job assignments
‘How did you get this job at the University?’ ‘What are your job assignments at the University’. ‘What do you like about the job at the University’ ‘What is difficult about the job at the University?’ ‘Do People at the University give you praise for the job you are doing here? ‘Have you ever been criticized for the job you are doing here?’ ‘Who do you turn to if you need help at work here?’ ‘Can you make your own decisions at work here? ‘Can you influence what job assignments you have here?’
3 Equality
‘Do people at the Department say hello to you when you arrive at work in the morning?’ ‘Do you feel that your work here is just as important as the work of the other employees?’ ‘What is it like to be with your colleagues at the University?’.
4 Communication
‘Do you talk to your colleges during the work day at the University? (When, where and how)?’ ‘Do you talk more with somebody in particular?’ ‘What is your lunch break like at the University?’

### Data analysis

All interviews were audio recorded and transcribed word by word. The data analysis was performed in accordance with thematic analysis (36). The process of analysis was inspired by the systematic text condensation method (37).

In step one of four in the text condensation method, the importance of openness is emphasized, listening ‘to the voice of the informants’, thus ignoring personal prejudice (37). Repeatedly reading the interviews separately, the researchers tried to familiarize themselves with the raw text before sharing their impressions with the other researchers. Subsequently, the researchers shared their thoughts and suggestions of preliminary themes. This early process was focused on developing an overview of interesting themes that had emerged like background experiences, job expectations and the person-activity match.

In the second step, this method has a focus on moving from themes to codes by finding and marking relevant text in a systematic review, sorting and organizing the data (37). Working first individually and then together, the researchers highlighted sections of text, phrases or sentences of relevance and compared sections that seemed related to each other, looking for codes to describe their content but also looking for relationships between different codes.

The third step involved a condensation process aimed at compressing the material into code groups by focusing on words or expressions that the informants had used in the interviews (37). Comparing the codes, the researchers gradually discovered patterns, and aiming at further condensation, the codes were organized into groups thus forming main themes. Subsequently, the researchers sought to formulate theme names that embraced the content of the individually worded codes. For an overview of the categories see **Table 2**.

**Table 2:** Categories.

Main categories	Sub categories
Experienced employees	Variation Motivating aquaintances
Suitable work challenges	Just right - difficulty of assignments - degree of autonomy Practicing acquired competences
Social belonging	Expanding ones network Common interests Being recognized Being appreciated Being respected

The challenge of step four in the analytical process, is to move from condensation to contextualization, by drawing on concepts, theories, and prior research (37). Consequently, the researchers tried to contextualize the emerged themes by reading up on and involving concepts, theories and prior research that might be of assistance in understanding our data and integrating them within the frame of prior knowledge. During this stage of the process, the researchers realized how much the participants focused on the social experiences related to their work assignments in the Department, and how these experiences tied in with the research literature on the role of belonging in inclusion and participation processes.

## Results

### Overview

The data generated many topics and possible categories. The researchers focused on the topics given most attention by the participants. The three main categories emerging from this analysis were: ‘Experienced employees’ (sub-categories: Variation, Motivating acquaintances), ‘Suitable work challenges’ (sub-categories: Just right, Practicing acquired competence), and ‘Social belonging’ (sub-categories: Expanding One’s network, Common interests, Being recognized, Being appreciated, Being respected).

### Experienced employees

Talking about background experiences, all the participants described their prior involvement in various forms of supported employment, thus making them ‘Experienced employees’ in the sense that they had practiced and tried out various types of work assignments and were used to trying out new jobs. One participant described it like this:

Before I did knit jobs and now, I am on fruit delivery /…/ and the canteen two days a week.

Trying out new jobs, they also made discoveries about what they liked to work with and which limitations they perceived. For instance, when one of the participants experienced that cleaning offices five days a week took a toll on her back, the participant had to make a change and started to work on transporting fruit some days and with cleaning other days. Now the participant had taken on one day a week at the University, expressing that the ‘Variation’ was important. Others had tried out jobs that turned out to be too demanding due to insufficient follow up. One participant had worked in a commercial kitchen and later in a shop and had similar experiences with both jobs. The participant described the shop experience as follows:

It was exhausting but I liked it there. It was hard work, a lot to memorize. It was too much for me to remember. I needed more help, closer follow up.

The participants seemed to describe that matching the person with the suitable job challenge had been important for their present well-being and prior job satisfaction. Furthermore, there was one criterion that almost all of them spontaneously reported when asked why they accepted the job at the University, and that was ‘Variation’. Being ‘Experienced employees’ they described having encountered alternative experiences in other work arenas and thus had developed preferences and wanted their working week to include some ‘Variation’. One of the participants formulated her thoughts when offered the job at the University like this:

Yes, that would be cool to try something new, something other than being at (name of supported employment firm). Getting away from that job a bit and being here a bit

Being at work five days a week was experienced as demanding and sometimes a bit boring for some of the participants, and thus novel experiences were described as attractive and exciting and a break with everyday life. Nevertheless, taking on a new job assignment working with strangers in a new arena could also be demanding and scary when not knowing what to expect. Therefore, some of the participants found it helpful to have some kind of connection to this new job arena. Being ‘Experienced employees’, some had learned that knowing someone at the workplace was useful, especially at the beginning. Thus, the second subcategory is called ‘Motivating acquaintances’. Some participants, when asked why they accepted the work assignment at the University, described having acquaintances at campus or that their parents knew somebody who worked there, or they had met with students in the Social Education Department when the students had practice periods in health and social services. Thus, some felt a connection to the University campus that made them more motivated to take on work assignments there. Summing up, the participants described coming to the University with prior knowledge of various job assignments and of how to adapt to new work arenas. The five participants reported knowledge that working life could offer ‘Variation’ and that being acquainted with people already working in the new arena might constitute ‘Motivating acquaintances’ and therefore had eased the decision making when offered a new job and making them even more ‘Experienced employees’.

### Suitable work challenges

In the process of finding work assignments for the new employees, worries emerged among the fulltime employees concerning what would be a suitable work load and type of work assignment. Consequently, several interview questions focused on how the participants experienced their work assignments. The five participants were unanimous in that they liked their work assignments and that the work load was ‘Just right’. To make sure that they were not just being polite about it and were afraid to complain or criticize their new employer, the researchers approached the topic from different angles, but the answers were consistent. One of them formulated it like this, when asked about the work assignments:

I like them very much

The researchers also tried to tap into the potential experience of boredom or overstimulation when asking if the tasks were easy or difficult, but they all described the tasks as easy and some explicitly expressed contentment that the tasks were not too difficult. One participant said the following about the work assignments:

I think they are quite easy to perform. But that is a good thing.

They all underscored that they did *not* want more difficult tasks and that they were very content with the tasks they had. They used expressions like ‘fun’ and ‘not boring’ and ‘not too difficult’ when they described how they experienced their work at the University. They said that the work load was ‘Just right’ and that they liked having sufficient time to execute the tasks without being stressed. One of the participants had not made the coffee that morning and commented that sometimes it was challenging to find the time to do all the tasks. The participant described sometimes ‘getting stuck’, explaining that being organized and getting things done could be challenging. Therefore, the participant remarked, it was important not having to hurry too much. To help the employees to get an overview of their tasks and keep organized, the contact person at the Department made a list over their regular tasks and tasks suggested by the staff as potential work assignments, and things they would like to have some help with that particular day. Several participants commented that the list was a good idea. They could carry the list along as they moved around the Department and thus get help to remember the program of the day. Consequently, when reading and analysing the descriptions of their work load and the challenge of their work assignments at the University, the subcategory ‘Just right’ emerged as that was an expression that the participants often used.

Although all participants were quite satisfied with their work assignments in general, one participant suggested the Department could draw on the participant’s experience as a cleaner to help clean offices:

However, it would be cool to clean offices, because I have never done that. To go from cleaning tables to cleaning offices. I would have liked to try that.

Unfortunately, such an assignment would interfere with the jobs of the full-time cleaning staff, which precluded exploring such work assignments. Even though the employer was unable to accommodate this particular wish, the participant expressed liking the present tasks also. ’Practicing acquired competence’ turned out to be a priority with the other participants as well. One participant was assigned the task of preparing and serving fruit and treats for the late Friday break, a popular event at the Department. The participant reported that:

I think it is fun to prepare the fruit, because I have done this kind of thing before, I took Catering and Hospitality in high school

These two participants expressed explicit ambitions, indicating that as they were ‘Experienced employees’ they had developed competences they wanted to practice and preferences about what kind of work assignments they would like. To accommodate these preferences, when possible, the Department as the employer, tried to find a good person-activity match. One example of such person-activity matching was when one of the participants demonstrated competence related to technology, he was offered tasks like checking printers for paper and ink cartridges. He helped staff operate the printer equipment and he also performed some maintenance on the printers. He commented on his work assignments saying:

I check on all the printers and then I check if there are some jobs left on the list (from the contact person). /…/ To be honest I really like it here

Consequently, the participants reported that they had some influence over their work assignments. They organized their work days together with their contact person in the beginning and gradually became more autonomous. Accordingly, they described even the amount of autonomy as ‘Just right’. When asked if they wanted to decide more for themselves at work at the University, one participant formulated it like this:

Not really

Summing up, the five participants communicated through the interviews an unequivocal experience of ‘Suitable work challenges’ in the form of ‘Practicing acquired competence’ and enjoying a degree of autonomy, type of work assignment and work load that was ‘Just right’.

### Social belonging

Many of the interview questions focused on their interpersonal relationships at the Department. However, sometimes participants spontaneously offered their experiences with their new work environment, such as when the researchers asked about the work assignments and if they sometimes were boring, one participant answered:

No, I just think it is fun to see you again and be together with you all.

The participant elaborated and said it was important because then there would be more people to talk to, not confined exclusively to those at the other job in sheltered employment, clearly indicating the need to ‘Expand the network.’ Two participants specifically talked about meting colleagues outside the work place, for instance at the supermarket, at the cinema and at Facebook, getting likes and have-a-nice-weekend-greetings. This is another example that ‘Variation’ for the five participants was underscored as important not only when it came to work assignments but also when it came to socializing. Furthermore, several participants found it to be ‘fun’ and ‘nice’ having lunch at the Department, ‘Sharing experiences’ and one particularly liked that the colleagues asked interesting questions like what had happened during the weekend. Another participant said:

I’m particularly fond of people here actually. All the people here. They are really kind and nice to talk to during lunch

Talking together during lunch was mentioned by several of the participants as an important social setting, something they appreciated and where they came to know their new colleagues. Since the participants had re-joined their work at the Department shortly after the Covid-pandemic, some focused strongly on the pleasure of being with other people and seemed particularly aware of how much this meant to them. One participant described the importance of ‘Common interests’ and how he talked about football during lunch, and that one of the participant’s colleagues had taken the initiative to display a collection of stones the colleague had in his office, since they both were interested in geology. Another participant brought up the importance of ‘Being recognized’ and paid attention to, like when the participant came back to work after the Covid-pandemic and her colleagues at the university said:

Ahh there you are A(name)

Thus, being missed when they were absent and welcomed back when present and being greeted by colleagues in the morning, were described by the participants as experiences they had appreciated. When asked explicitly about how they were met and treated when at work at the university, the word ‘kind’ was used by most participants to describe the attitude of their colleagues. One participant added that the colleagues were kind, and also so to the others from sheltered employment settings, indicating that they had shared their experiences of working at the University, agreeing that the colleagues were nice to them. One participant pointed out he had always been a loner but still enjoyed lunch at the University and talked to some colleagues although maybe not as much as others, but for this participant it was ‘Just right’. One colleague was of particular interest to the participants and that was their contact person at the Department. They described their colleague’s presence as essential to their adaption process in a non-sheltered environment and pointed to their colleague’s sense of humour as helping them feel accepted and at ease. Several participants pointed out that he helped them so they could keep track of their assignments, and was present to answer questions, which was important since many of their colleagues where busy during working hours and at times kept their doors closed and could not be contacted.

The participants also described ‘Being appreciated’, getting praised and feeling that the job they did was important. After Covid, some participants often got spontaneous positive comments about cleaning the door handles with high alcohol sanitizers. One of them said this was a proper job because it was different from just sitting at home and that was the important thing. When asked whether the job at the University felt important another participant replied:

Well, it’s my coffee they are drinking…

Most of the participants explicitly expressed that being able to say that they worked at the University made them proud and the reaction of other people gave them a clear understanding that working at the University was an important job that other people respected.

Summing up, the participants described several types of appreciated social experiences during their time at the University. Some participants ‘Expanded their network’ and got more ‘Variation’ in their social life, others found ‘Common interests’ with particular colleagues, and almost all explicitly described the importance of ‘Being recognized’ and ‘Being appreciated.’ Moreover, the way they described their being with their colleagues the experience of ‘Being respected’ could be added.

## Discussion

The voices of people with ID are often missing in debates on how to promote social inclusion (1, 3). Consequently, it is important to learn more about how people with ID themselves experience their exploration of community participation in general and inclusion in non-sheltered employment in particular. Trying to promote social inclusion in the community, the Department of Social Education at the Arctic University of Norway (UiT) offered customized work assignments to 5 employees with ID. This project was aimed at including people with ID in higher education, a high-status arena from which they were totally absent but that might offer them new and interesting experiences. During their first year at UiT, these employees gained many experiences related to social inclusion which they shared in qualitative research interviews, among the most prominent experiences that emerged were the importance of becoming an ‘Experienced employee’, developing ‘Suitable work challenges’ and experiencing ‘Social belonging’.

### Experienced employees

When reading the interviews, the first thing that surprised the researchers was the participants’ descriptions of their varied experiences with work. During the recruitment process the researchers had learned that the participants presently were employed in sheltered employment. In Norway, only one of four adults with ID are employed and almost exclusively in sheltered work (38), indicating that there is a limited freedom of choice when it comes to types of work available. Although new governmental strategies encourage the transitioning of more people with ID into non-sheltered employment, such a development has not been achieved so far (39). The problem of finding work for people with ID has also been described in studies from Australia and Sweeden, where young unemployed adults with ID subsequently reported feeling frequently bored (3, 40).

Nevertheless, the participants in this study described having explored three or four different types of work assignments prior to present employment. Having had the possibility of experiencing various types of work assignments seemed to have made them more self-reliant and therefore motivated to try something new, thus taking their chances on working in a non-sheltered environment, finding the courage to trust and cooperate with their new employers to customize a suitable job for them. This is especially important for employees with ID as their career choices are often influenced more by their environment than by their own preferences (41). Studying employment sustainability in people with ID, one study (40, p. 78) showed that ‘having tried various types of work’ was one of five facilitators of employment sustainability. They argued that this kind of experience was particularly important for adults with ID, because they would have more difficulties *imagining* what various jobs were like and therefore hands on experience might help them make better decisions in the future. In accordance with this line of thought, the participants in this study described how having tried various jobs made them more aware of their own preferences and their capacities, making the exploration of working in a non-sheltered environment seem more manageable. Based on their descriptions, experiences with sheltered employment seemed to constitute an important base for their transition into a non-sheltered work environment. It must be added here that the sheltered employment described by the participants consisted of activities that were both work related and age appropriate. These findings are in accordance with (42, p.6) the concluding remarks of other researchers after reviewing studies on employment outcomes for people with intellectual and developmental disabilities, commenting that sheltered employment ‘serves as a strong foundation for providing job skills that assist such individuals in moving into a mainstream environment with integrated and competitive employment.’ Thus, the participants seemed to have acquired important skills through becoming ‘Experienced employees,’ skills that under the right circumstances may contribute to community participation and social inclusion by working in non-sheltered environments. This might indicate that less experienced employees might need other kinds of pathways into non-sheltered environments for example through a more comprehensive support system.

Having tried different types of work assignments prior to their present jobs, several participants had experienced the importance of ‘Variation’. Even though they spoke positively of their present jobs in sheltered employment, several participants explicitly expressed that they found doing something different one day a week was appealing. One of them stressed the importance of meeting new people and having a chance to expand the social network. Another participant focused on the importance of having a job at all, underscoring the significance of having somewhere to go and something to do to avoid boredom, monotony passivity and feeling isolated. Seen in the context that studies involving adults with ID in different countries have reported their experiences of feeling ‘left out’, having trouble finding employment, little variety of opportunities and being frequently bored (3, 40, 43), work related activities in sheltered employment seemed important to our participants by giving them a chance to discover the importance of variation. Regarding being ‘Experienced employees’, most of the participants in our study described *explicit aspirations* to explore various possibilities to eventually find meaningful employment through ‘Suitable work challenges’ also involving taking the risk on being successfully included in non-sheltered environments.

### Suitable work challenges

To achieve social inclusion in a new arena, finding suitable work challenges for the new employees at the Department was a primary goal. This task had already activated worries and discussions during the planning process of this inclusion project. Considering the worries expressed by the full-time employees, the participants expressed a surprisingly high degree of contentment when asked about how they experienced their work challenges. All but one of the participants underscored that the challenges were ‘Just right’ and some even indicated that they were more worried about getting work assignments that were too difficult than about being bored. Interestingly, another study (44) also found that when people with ID reported on their experiences with starting a new job, some shared their concern that their competencies might not meet the demands of their employers. These results may contribute to an explanation of why three of the participants expressed explicit appreciation of the adjustments made to take their prior competencies into account when customizing their job assignments to find the challenges that were ‘Just right’.

However, even the participants who found potentially interesting work challenges that were not available for exploration due to administrative regulations, did not express discontent with their actual work challenges. Being ‘Experienced employees’ one might speculate that they had prior knowledge of such restrictions in sheltered working life, thus being aware that autonomy in working life is relative and is practiced within the limits set by the administration. Reviewing studies on feelings about work in people with ID, the researchers (44) found that although some people were disappointed with the routine nature of their assignments, they also recognized that some work assignments were beyond their abilities, indicating that with experience comes a certain understanding of one’s own capacities and what is a suitable challenge. Nevertheless, what is a suitable challenge at a new job may become a monotonous work assignment later on. One study (40) showed, looking at employment sustainability in people with ID, that two factors were of particular importance. First, the personal characteristics, particularly their ability to learn and develop, are central. This result seems particularly relevant to the current study, as the participants described substantial flexibility in trying out various jobs, being willing to learn new skills and adapt to new work environments. It is this *willingness to develop* that is crucial to finding the suitable and sustainable work challenge, according to this particular study (40). Taking advantage of the participants’ willingness to develop might be a challenge for the further success of the inclusion project explored in the current study. As the participants become more experienced with their work assignments and the excitement of a non-sheltered work environment wears off, following up on new demands on more challenging or competence-related work assignments might exceed the limited resources so far available in the project. One of the participants already had expressed a preference for cleaning offices instead of cleaning the kitchen and tidying the lunch room and was turned down due to work life legislation and labour union policies.

Second, one study found that employment sustainability was influenced by circumstances surrounding the individual, such as co-workers’ and managers’ attitudes and behaviours, including employer’s flexibility (40). In the current study, the participants described the managers’ attitudes to be positive, allowing them the ‘Just right’ amount of autonomy, involving them in finding and designing ‘Suitable work challenges’, giving them a list of assignments to choose from and in particular seeking out work assignments enabling them to practice acquired competencies. This flexible process has similarities with customized employment, described as a personalization of the employment relationship to meet the needs of both employees and employer (45). In the process of developing these ‘Suitable work challenges,’ the participants described how their contact person and co-workers were sufficiently available to them when they had questions or needed help so that they would not feel overwhelmed or insecure at work, ensuring that their needs were met. The importance of what in the research literature has been referred to as *natural supports*, that is support typically available and provided by work place resources, is well established (45).

Much of the support described by the participants in this study might be classified as *natural supports*, resembling what is given to all new employees, including having a support person called mentor during the first year at the Department. However, this support person worked closer and spent more time with the participants than the standard mentoring given full time employees. Moreover, little research has studied how to effectively provide support for employees with ID (45). Reviewing the literature, they concluded that accessing work place performance support was essential for successful employment outcomes, as was social integration. One factor was pivotal, namely the training of the support providers, as most formal employment support providers were well educated and experienced, which might have influenced their capacity in facilitating support. The training of support providers is particularly relevant for this study, since all the support providers were well educated and had prior experience in working with people with ID. Accordingly, this might partly explain why the participants described to have received the appropriate level of support they required during their first year of employment in a non-sheltered environment.

Nevertheless, keeping up the effort and the commitment to work actively with including the new employees may become more of a challenge with time. As the staff become adapted to their new colleagues these may receive less attention and be left to fend for themselves. Most new projects tend to lose some interest over time, particularly as many universities are experiencing an increased work load and budget cuts. This situation may also influence the resources available to uphold the natural support reported to be particularly important to the well-being of the participants at work.

### Social belonging

So far, the discussion has covered the importance of the participants characteristics as ‘Experienced employees’ and how these experiences had constituted a basis for them in successfully negotiating ‘Suitable work challenges’ in their new work environment. However, the participants had joined a non-sheltered work environment at the University something with which they had limited prior experience. Although they had interacted with non-sheltered work environments while employed in sheltered work for example when delivering fruit or laundry, this interaction had not included the more extensive collaboration and affiliation involved in working a full day in a non-sheltered work environment.

Looking at the reports of the participants on the social interaction with other employees at the department, there is little if anything to indicate experiences of discrimination, rejection or isolation. The participants described their social interaction at work in very positive words like people being ‘kind’ and ‘nice to talk to’, they describe being together as ‘fun’ and say they are ‘being fond’ of everyone there. These experiences are of particular importance to answer the question of mere physical presences vs actual social integration, as not feeling accepted has been one of the main factors identified as negatively influencing community participation and inclusion (27). Asking people with ID themselves about integration factors they named *lack of acceptance* and *negative attitudes* among the essential barriers (1). The descriptions of social interactions given by the participants in our study unanimously communicated beneficial experiences with acceptance and positive attitudes. This absence of the negative attitudes found in several other studies (27) is mostly likely related to the composition of this particular group of employees. The staff was mostly social educators having worked with people with ID, teaching about disability in general and ID in particular. Some employees were psychologists, sociologist or special education teachers all with a professional interest in understanding and improving the lives of people with intellectual and other disabilities. The staff ‘s level of knowledge, ethical and political engagement in ameliorating the lives of people with ID, is a likely contributing factor to the positive and accepting working environment described by the participants. However, it is also likely that being ‘Experienced employees’ the participants were offered the work at the university because they had proved to be socially competent and therefor were more likely to succeed in a non-sheltered environment. It has been argued that people with high social capital are more likely to obtain employment and score higher on measures of integration (7).

The participants described how they were involved in personal conversations with staff during lunch and other social events and experiencing the level of autonomy and work challenge as ‘Just right’. These descriptions indicate that they felt respected and treated like adults and at the same time getting the right amount of support particularly through their mentor/support person. In another study of young adults with ID the participants described acceptance as people initiating conversations with them and treating them with respect like any other person (29). Thus, acceptance and respect are described by people with ID as important aspects of social belonging. Nevertheless, this is not sufficient to activate an overall feeling of belonging, according to some (16), belonging also includes being present, invited, welcomed, known, accepted, supported, heard, befriended, needed and loved. Looking at the descriptions from our participants they do report being present, being recognized and greeted every day at work, being invited to participate in various social events and conversations, coming to know their colleagues, some better than others, and feeling needed when they performed tasks that the staff explicitly has expressed that they want help with. However, one year into the project the participants had not befriended any of the staff in the sense that they met regularly outside the department. What they did describe was meeting people from the university outside the University, where they stopped and chatted, and several were friends with staff members on Facebook and communicated regularly with them there. This is a level of contact that is equal to what many other staff members have with their colleagues at the Department.

Nevertheless, it seems appropriate to keep in mind that the participants in this study were relatively socially outgoing. The reason they were ‘Experienced employees’, may partly be because they had good social skills and thus were chosen for various job assignments due to these skills, as their supervisor in sheltered employment thought they had better chances of succeeding in contact with non-sheltered work environments. Furthermore, the researchers interviewed fellow employees and that might have made criticism and reporting negative experiences difficult for the participants. To accommodate this challenge, the researchers spent time explaining how negative experiences were important to the University so that the staff could improve their effort in facilitating their transition to the University. On the other hand, having been introduced to the participants prior to the interviews also made the interviews seem less scary and made the conversation more fluent. It is possible that some negative experiences were not reported, however, the spontaneity and enthusiasm with which they communicated their experiences of belonging does indicate that this was their main experience.

Summing up the discussion, it seems that the participants involved in this inclusion project at the University, reported in accordance with many other studies (23) that they liked working in a non-sheltered environment. In line with prior research (40, 42) they also described how their experiences with sheltered employment helped them to seek and master non-sheltered employment. Natural support has also been found to be essential for people with ID to succeed in non-sheltered employment (45) and participants in the current study explicitly described how accessing natural support during their working day was an important factor in promoting their well-being at work at the University. Finally, according to prior research, negative attitudes and lack of respect have been reported to be some of the most important barriers to succeeding in non-sheltered environments (1) and most of the participants in the current study stressed experiences of ‘Social belonging’ as the central factor for their happiness at work. However, when it came to preferring non-sheltered over sheltered employment, the participants in the current study were not as clear in their preference as suggested in a recent review (23).

The current study is based on the experiences of 5 participants, thus making it difficult to make strong recommendations from these data alone. Nevertheless, it seems that data from the current study suggest that higher education can be a well-suited arena for inclusion projects helping people with ID transmission into non-sheltered employment provided the participants are offered the necessary support. Subsequently, according to existing policies and mission statements, the University’s future plans ought to include more projects involving people with various disabilities.

### Limitations

The main aim of this study was to explore the subjective experiences of people with ID being employed in a non-sheltered work environment in higher education. Although the participants constituted a small sample and were present at the University only one day a week, the researchers found it relevant to interview them because there are few studies describing the experiences of people with ID working in the arena of higher education, partly because they constitute a small population. These interviews were also aimed at studying how people with ID experienced their transition into a non-sheltered work environment. There are clear limitations to a study based on such a small sample and these findings cannot be generalized to large groups. Nevertheless, qualitative research is defined as ‘the study of the nature of phenomena’, including ‘their quality, different manifestations, the context in which they appear or the perspectives from which they can be perceived’ (46). Thus, the contribution of this study was to describe one such example of inclusion and the context in which it appeared.

Communicating with participants in research interviews is always a challenge, trying to interpret other people’s statements is complicated and misunderstandings are a known source or error in qualitative studies (37). Communicating with persons with ID added a few challenges. Making sure the researchers had understood what the participants were saying the way they meant it, was given extra attention. Thus, the researchers did member checking with the three participants who gave their consent to a second interview. Ideally, all the participants should have been re-interviewed to ensure that our interpretations of the communication would be as close to the intended message as possible.

The researchers first asked open questions about the participants’ experiences with their colleagues. Second, the researchers attempted to make negative answers as normal and acceptable as positive answers, as it is difficult to openly criticize people at work. However, none of the participants used any of the suggested answers but replied in their own words. Another factor that might influence the results of the study was that although all the participants had several prior experiences with sheltered employment, some participants had more long-term experiences than others and it is possible that these variations could impact the study findings.

The main limitation of this study is probably the small sample and recruiting participants from only one department. Nevertheless, these participants constituted all the people with neurodevelopmental disorders (most of them had ID) employed in this inclusion project. This limitation of small samples may be difficult to remedy in the near future since not many people with ID are employed in non-sheltered employment at universities and that does not seem likely to change in the near future. However, performing more long-term in-depth studies on small samples may still contribute useful knowledge about how people with ID can enter into, adapt to and learn to navigate such new non-sheltered work arenas.

## Conclusion

In this study, the researchers aimed to examine the experiences of people with ID participating in an inclusion project at a University. The researchers were particularly interested in exploring if their *presence* in this non-sheltered work environment promoted experiences of social inclusion through the feelings of belonging, as this had been highlighted as a mark of successful inclusion.

In the interviews, the participants described being satisfied with their work challenges at the University. However, what was by the participants described as suitable working challenges at the time of the interviews, could later be experienced as lack of variation, especially since the participants described their present challenges as well within the borders of their competencies. This may be an upcoming challenge for the inclusion project as the participants become more accustomed to their new work arena. Being ‘Experienced employees’, the participants demonstrated an eagerness to learn and embrace ‘Variation’ and change.

Nevertheless, the participants in this study did describe the development of positive relationships as a main source of well-being and motivation in their transition to a non-sheltered work environment. Bearing in mind that prior research has found most people with ID to have rather fragile social networks, the increase of social experiences described by the participants, although limited, seemed to indicate an expansion of their social network that mattered to them, making them feel invited and connected thus describing experiences of belonging as defined in the research literature. The participants’ descriptions of belonging also met the criterion, that community exposure must be experienced as beneficial to the participants thus indicating that their integration in a non-sheltered environment had been successful in that respect. However, in the research literature the discussion is often focused on the pros and cons of sheltered and non-sheltered work inclusion giving the impression that one or the other have the most advantages and thus must be preferred. Nevertheless, the participants in this study reported that it was the *variation* of being both in sheltered and non-sheltered work environments that was satisfying to them. They had their friends and their secure jobs at PAW and at the same time they were challenged one day a week by being in a non-sheltered work environment where they could explore new experiences, expand their network and learn about non-sheltered working life. They reported this combination to be ‘just right’ for them.

## Data Availability

The datasets presented in this article are not readily available because of Norwegian regulations and privacy concerns. Requests to access the datasets should be directed to gro.h.ramsdal@uit.no.
